# Cryptococcal antigen positivity combined with the percentage of HIV-seropositive samples with CD4 counts <100 cells/μl identifies districts in South Africa with advanced burden of disease

**DOI:** 10.1371/journal.pone.0198993

**Published:** 2018-06-12

**Authors:** Lindi-Marie Coetzee, Naseem Cassim, Charlotte Sriruttan, Mabatho Mhlanga, Nelesh P. Govender, Deborah Kim Glencross

**Affiliations:** 1 National Health Laboratory Service (NHLS), National Priority Programmes Unit, Johannesburg, South Africa; 2 Department of Molecular Medicine and Haematology, Faculty of Health Sciences, University of Witwatersrand, Johannesburg, South Africa; 3 National Institute for Communicable Diseases (Centre for Healthcare-Associated Infections, Antimicrobial Resistance and Mycoses), Division of the National Health Laboratory Service (NHLS), Johannesburg, South Africa; 4 Faculty of Health Sciences, University of the Witwatersrand, Johannesburg, South Africa; Centers for Disease Control and Prevention, NAMIBIA

## Abstract

**Introduction:**

Cryptococcal meningitis (CM) is an opportunistic fungal disease with a high mortality among HIV-positive patients with severe immunosuppression (CD4 count <100 cells/μl). Reflexed screening for cryptococcal antigen (CrAg) in remnant blood samples was initially piloted at selected CD4 testing laboratories of the National Health Laboratory Service (NHLS) prior to the implementation of a national screening programme using a lateral flow assay (LFA) (IMMY, Norman, OK, USA). The aim of this study was to assess CrAg positivity nationally, per province and district in combination with the percentage of CD4 samples tested with a CD4 count <100 cells/μl to identify areas with advanced HIV/CrAg disease burden.

**Methods:**

CrAg and CD4 laboratory result data were extracted from the NHLS corporate data warehouse. Monthly test volumes were used to assess CrAg test volumes and coverage, while bubble charts were used to display the relationship between CD4 <100 cells/μl, CrAg positivity and number of positive CrAg samples by district. ArcGIS software was used to spatially report CrAg positivity.

**Results:**

CrAg screening coverage was stable at around 96% after November 2016. Samples with a CD4 <100 cell/μl and CrAg positivity were also stable over the study period at 10% and ~5% respectively. The highest CrAg positivity was reported for the Kwa-Zulu Natal province (7.3%), which also had the lowest percentage of samples with a CD4 <100 cells/μl (7.2%). Uthungulu and Umkhanyakude districts had the highest CrAg positivity (9.3% and 8.9% respectively). Ethekwini and Johannesburg Metro districts contributed to 22% of the total number of CrAg-positive samples tested across South Africa for the period reported.

**Conclusion:**

Existing CD4 testing services were used to rapidly scale up CrAg reflex testing in South Africa. Districts with advanced HIV and CrAg disease burden were identified that need further investigation of patient management interventions.

## Introduction

Cryptococcal meningitis (CM), caused predominantly by *Cryptococcus neoformans*, is an important HIV-related opportunistic infection particularly among immunocompromised patients in developing countries including South Africa [1–3]. The global CM incidence estimate is 223 100 cases per annum, with Sub-Saharan Africa reporting the highest incidence (162 500 cases annually) [4]. CM morbidity and mortality is particularly high in low and middle-income countries (LMIC) with concomitant high HIV prevalence [4–7].

Despite the availability of antiretroviral therapy (ART) in South Africa, approximately 10% of HIV-infected patients still present to care with a CD4 count <100 cells/μl [8]. Patients with advanced HIV disease, defined as CD4<200cells/μl as per World Health Organization (WHO) guidelines, are more vulnerable to CM which is associated with high mortality rates even with efficacious and prompt antifungal therapy [6]. While early diagnosis of HIV infection and initiation of ART prior to the development of AIDS is critical to reduction of CM incidence, a cryptococcal antigen (CrAg) screen-and-treat intervention has the potential to reduce cryptococcal disease-related mortality by identifying patients prior to onset of CM [6, 9]. CrAg screening is a cost-effective intervention, even at a very low prevalence.

Reflexed laboratory-based CrAg screening enables automated testing of remnant CD4 samples with a confirmed count below 100 cells/μl, using the manual lateral flow assay (LFA) [10] (Immuno- Mycologics, IMMY, Norman, OK, USA). This facilitates the simultaneous reporting of both a CD4 count and a CrAg result, to ensure prompt clinical intervention if CrAg is detected in blood [11, 12]. To assess the feasibility of this approach in South Africa, a pilot laboratory-based CrAg screening programme was initiated in 2012 [13] across four National Health Laboratory Service (NHLS) CD4 testing laboratories. This led to the implementation of a national CrAg reflex screening programme in June 2016 with approval from the National Department of Health (NDOH) [14, 15], with all CD4 testing facilities providing this service by October 2016.

In order to assess the coverage of CrAg testing (i.e. CrAg samples tested as a percentage of the total number of CD4 samples with a count <100 cells/μl), manage service (using operational indicators) and provide for future service planning (identifying gaps in current CrAg testing services), a thorough review of all CD4/CrAg data from the NHLS corporate data warehouse (CDW) was conducted. Specimen CrAg test data across a network of CD4 laboratories can provide valuable population level insights into CrAg burden across the country. This study builds on previously published data that identified districts for intensified patient management using the percentage of CD4 counts <100 cells/μl as test parameter [8].

The aim of the current study was to report the relationship between advanced HIV disease (percentage of CD4 samples with a CD4 count <100 cells/μl) and CrAg positivity per province and district across South Africa using operational laboratory specimen-level data.

## Methods

### A pilot CrAg prevalence survey prior to national implementation of reflex CrAg testing

A CrAg prevalence study was conducted from June 2014 to July 2015, at 12 pre-selected CD4 testing laboratories. These laboratories did not participate in the CrAg pilot programme (i.e. not performing reflexed CrAg screening at the time) and represented each of the nine provinces of South Africa. Remnant blood samples with a confirmed CD4 count of <100 cells/μl, collected over 5 consecutive working days and submitted to the National Institute for Communicable Diseases (NICD) for CrAg testing using the Immy LFA kit. CD4 absolute count, median and inter-quartile ranges (IQR) were calculated as well as the prevalence per province as represented by the selected testing CD4 laboratory.

### Data analyses after national implementation of reflexed CrAg screening

All 49 NHLS CD4 laboratories performed reflex CrAg screening using remnant plasma for all samples with a reported CD4 <100 cells/μl from October 2016 onwards. Samples were identified using the rules-based feature available on NHLS laboratory information system (LIS).

Two primary measures of occurrence are described in this study. Firstly, the number of CD4 samples tested with a count <100 cells/μl divided by the total number of CD4 samples tested as a percentage globally, per province and per district. The second measure describes the number of CrAg-positive samples as a percentage of the total number of CD4 samples with a count <100 cells/μl.

### Data extraction and analysis

CD4 and CrAg aggregated programme specimen-level data were extracted from the CDW for the period July 2016 to April 2017. Institutional ethics approval (M120124, M1706108; University of the Witwatersrand) was in place for unlinked anonymous data usage (no reference to patient information other than test results). The data extract included the episode number, date of testing, referring health-facility and province, health district, testing laboratory, CD4 count and CrAg result (defined as positive, negative or equivocal). Where a CrAg test was not performed for a CD4 <100 cells/μl sample, this result was categorized as “CrAg not performed” versus “CrAg performed”. The year and month values were extracted from the LIS reviewed date (date that the result was authorized for release by the testing laboratory) to compare test volumes per month through the implementation phase. Each health facility was defined in the CDW by their respective parent province and health district. Microsoft Access and Excel software were used to store and analyze data.

Data reported include the percentage of CD4 samples with a count <100 cells/μl and the percentage of CrAg-positive samples reported overall, per province and per district.

### Scale-up of CrAg screening

National scale up is represented by a month-on-month bar graph to illustrate increase in CrAg test volumes and national testing coverage (percentage of CrAg samples tested/all CD4 samples with a confirmed count <100cells/μl).

### %CD4 < 100cells/μl, CrAg positivity rates and number of CrAg-positive results by province and health district

Microsoft Excel was used to calculate CrAg positivity and the number of CrAg-positive samples at provincial and health district level and generate bubble maps for visualization of health district data. The number of CD4 samples <100 cells/μl as percentage of the total volumes of CD4 tested, was also calculated.

### Spatial reporting of CrAg positivity rates by health district

The district CrAg positivity was spatially reported as a map using ArcGIS software with color-coded bins at 2% intervals. The district shapefile was obtained from the municipal demarcation board (MDB). The Municipal Demarcation Board is an independent authority responsible for the determination of municipal boundaries of South Africa since 1998[16]. ArcCatalog software was used to edit the shapefile to capture the calculated CrAg positivity using the ArcMap editing feature. Data were independently checked for accurate transcription. Bins were created and colors assigned using the symbology function in ArcMap.

## Results

### Initial CrAg prevalence survey

1574 test samples were collected from 12 CD4 laboratories and tested for CrAg. Of these, 53 tested positive, i.e. an overall prevalence of 3.4%. The provincial prevalence of cryptococcal antigenaemia ranged from 1.3% in the Free State (FS) province to 6.5% in Kwa-Zulu Natal (KZN). A median CD4 count of 41 cells/μl (IQR of 21–57 cells/μl) was noted for CrAg-positive samples vs. a median of 49 cells/μl for CrAg (IQR of 25-74cells/μl) negative samples (p = 0.09).

### Scale-up of CrAg screening

**Fig 1:**
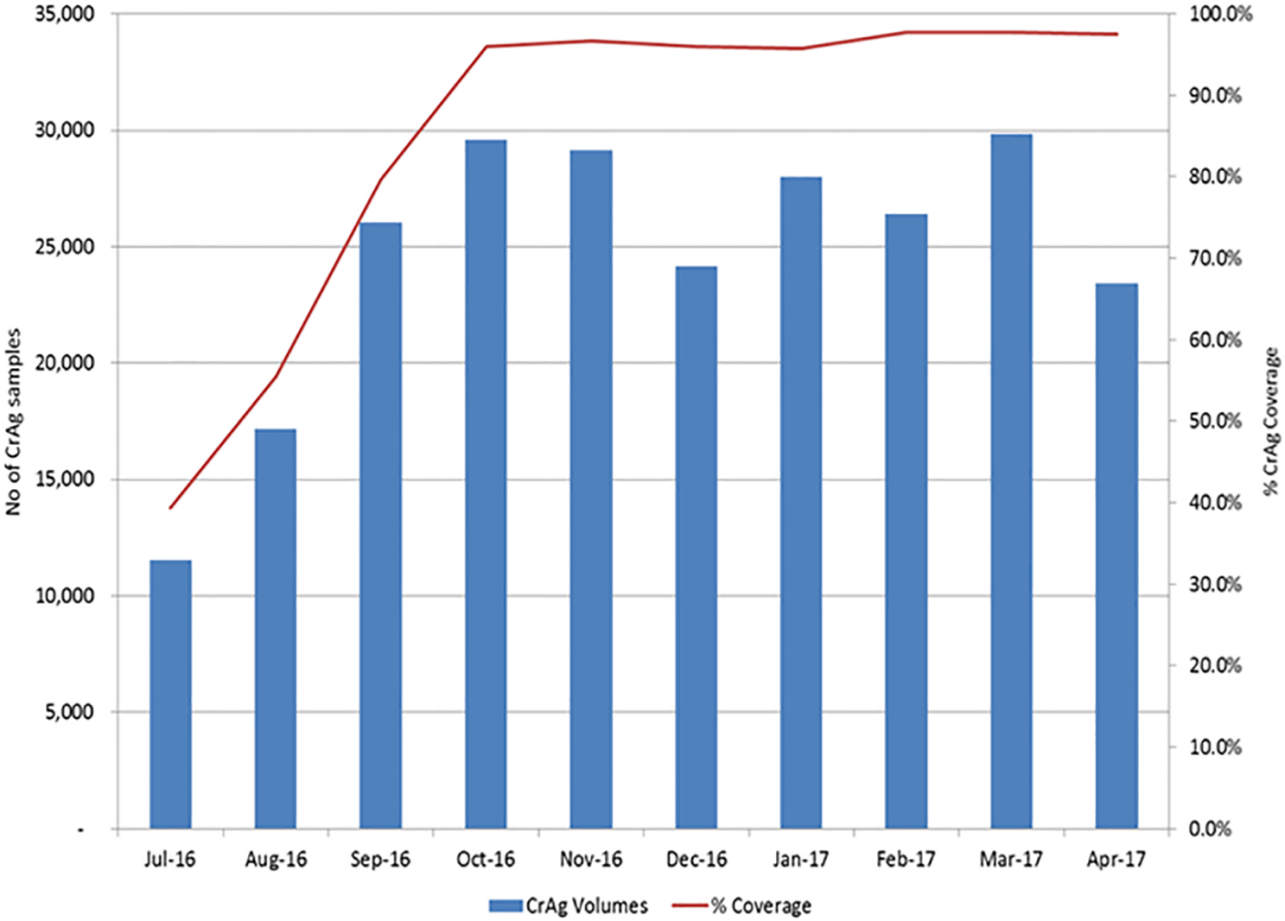
CrAg test volumes. Bar graph to report CrAg test volumes (blue bar) and percentage coverage (red line) for the test period June 2016 to April 2017.

The national scale up of CrAg reflex screening is depicted in Fig 1 for the test period.

By November 2016, coverage of CrAg screening services increased to 96.7% (n = 29135 tests) and stabilized at approximately this level for the test period. After November 2016, CrAg volumes followed the same monthly fluctuation patterns seen for CD4 test volumes (i.e. lower in December and April owing to public holidays), maintaining high levels of coverage and a monthly average of around 24 000 CrAg reflexed tests across 49 testing facilities.

Data reported here includes only reflexed CrAg screening performed through the CD4 laboratory network. CrAg testing performed through clinician-initiated requests using NHLS microbiology laboratories is excluded for reporting in this study.

### Categorization of provincial performance using proportion of CD4 <100cells/μl, % CrAg positivity rates and absolute number of positive CrAg samples tested

For the test period, a total of 245 270 CrAg tests were conducted, with test volumes ranging from 4361 to 65044. Gauteng conducted the most tests, followed by KZN and Eastern Cape. These three provinces also had the highest percentage of CrAg-positive samples, with KZN having the highest positivity (7.2% vs. national average 5.4%). Three provinces had a higher than national average (10.1%) incidence of CD4 samples with a count <100 cells/μl, namely Limpopo, Gauteng and North West provinces. These provinces had a corresponding CrAg positivity equal to (Gauteng) or below (Limpopo and North West) the national average of 5.4%. The Northern Cape processed the least number of samples with the lowest CrAg positivity (2.4%) and prevalence of CD4 <100 cells/μl (10.5%).

The majority of samples tested through the CrAg reflex programme for the reported period were referred from clinics (59.6%) vs. hospitalized patients (36.5%), with CrAg positivity of 3.9% and 7.9% respectively.

### Categorization of health district performance using proportion of CD4 <100 cells/μl, % CrAg positivity and absolute number of positive CrAg samples tested

**Fig 2:**
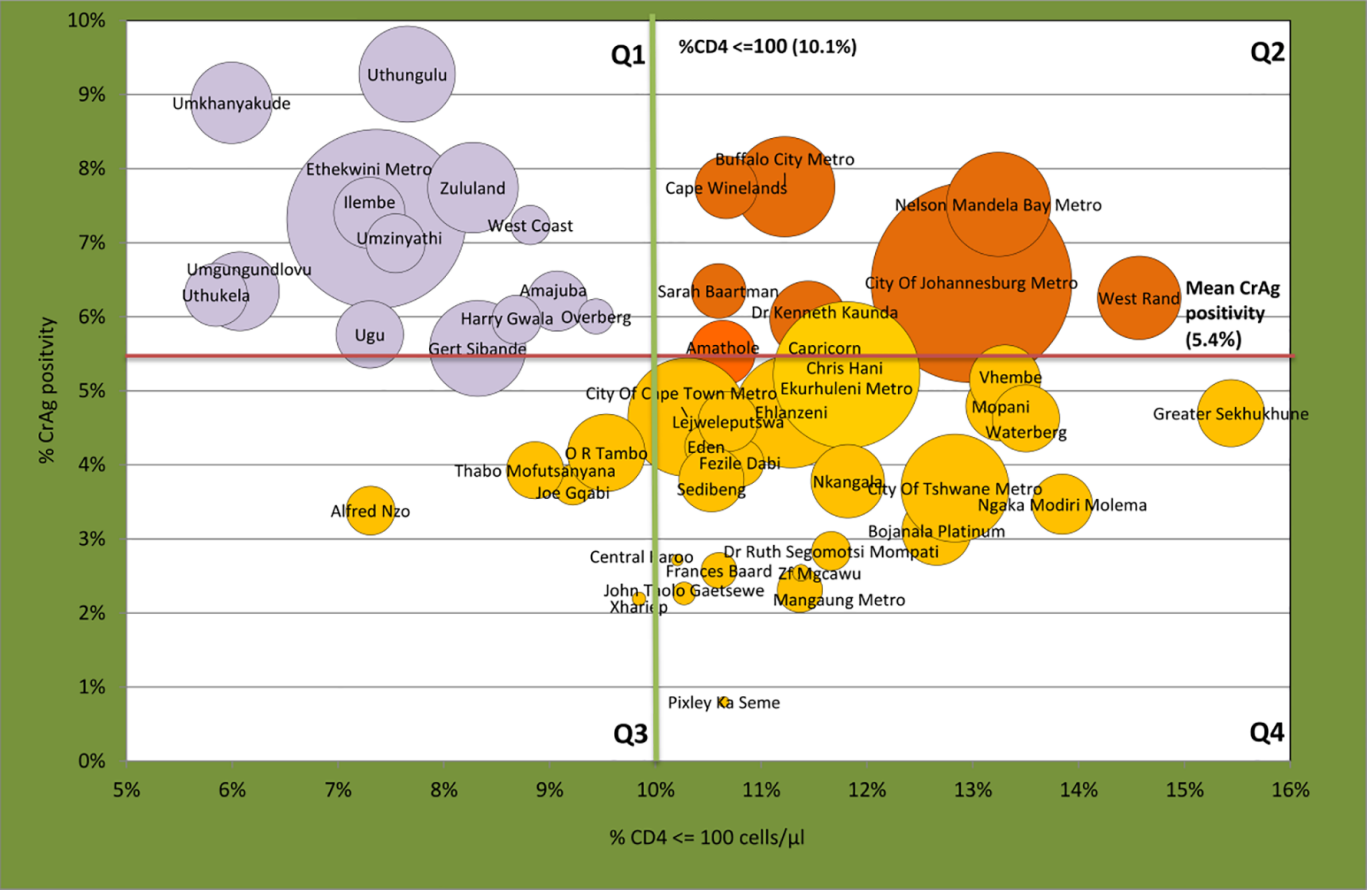
Summary of district data. A bubble map (linked data in the table below) showing the %CD4<100cells/μl (X-axis) vs. the % CrAg positivity (Y-axis) for 52 South African districts, where bubble size represents the absolute number of CrAg-positive samples reported. The solid green line represents %CD4<100cells/μl at 10.1% national mean, with the red solid line at 5.4%, representing the national mean CrAg positivity. Quadrant 1 (Q1; lilac) represents districts with high % CrAg positivity and lower than national mean %CD4<100. Quadrant 2 (Q2; dark orange) represents districts with higher than national mean CrAg positivity and percentage CD4<100, while Quadrants 3 and 4 (Q3 and Q4; light orange) represent districts with lower than national mean CrAg positivity with varying rates of %CD4<100 (Q3 below national mean and Q4 above national mean).

A health district bubble map was created in Microsoft Excel, reporting the % of CD4 samples with a count <100 cells/μl (x-axis), %CrAg positivity (y-axis) with the bubble size representing the absolute number of positive CrAg samples for the 52 districts. Four quadrants (Q1 to Q4) were created by the intersection of the national means for the proportion of samples with CD4 counts <100cells/μl and %CrAg positive samples (red and green lines) (refer to Fig 2 legend).

In line with the findings for Table 1, Q1 reveals predominantly KZN districts. The West Coast and Overberg districts from the WC also clustered in this quadrant (both coastal districts). Gert Sibande and H Gwala districts were also noted to cluster with this group and (geographically) border on the KZN province. These districts reported a higher-than-national mean CrAg positivity even though their percentage of CD4 samples with a count <100 cells/μl was below 10.1% (national mean). Of all the KZN districts, Ethekwini reported the highest number of CrAg-positive samples for the period reported (n = 1326). Uthungulu and Umkhanyakude reported the highest overall CrAg positivity (Fig 2, Q1, i.e. 9.3% vs. 8.9% respectively).

**Table 1:** Summary of provincial CrAg data. Data reported are in order of the percentage CrAg positivity per province, with reference to the percentage of CD4 samples with a count <100cells/μl and total number of CrAg-positive tests vs. all CrAg tests performed.

PROVINCE	% CD4 <= 100	% CrAg positivity	No of CrAg-positive samples	Total CrAg samples tested
Kwa-Zulu Natal	7.2%	7.3%	3,545	48,760
Eastern Cape	10.5%	5.7%	1,817	31,608
Gauteng	12.6%	5.4%	3,495	65,044
Western Cape	10.2%	5.1%	947	18,479
Limpopo	13.1%	5.0%	1,143	22,869
Mpumalanga	10.4%	4.7%	1,127	23,829
North West	12.4%	3.8%	659	17,138
Free State	10.3%	3.6%	476	13,182
Northern Cape	10.5%	2.4%	106	4,361
**NATIONAL**	**10.1%**	**5.4%**	**13,315**	**245,270**

In Q2, Cape Winelands (WC) and East-London Metro (EC) reported the highest CrAg positivity rates of 7.7 and 7.8% respectively (coastal areas). Other district outliers included Kenneth Kaunda (FS, geographically adjoining the West rand district of GP) and the Johannesburg Metro (GP), which reported the largest number of CrAg-positive samples (n = 1658), with a positivity of 6.5%.

For Q3 and Q4, the districts had a corresponding CrAg positivity <= 5.4%. Most of these were located in Q4, where the % samples with a CD4<100cells/μl was higher than 10.1%, with only 5 districts noted where CrAg positivity and %CD4 <100 cells/μl were both below the national mean values (Q3).

### Spatial reporting of CrAg prevalence by health district

**Fig 3:**
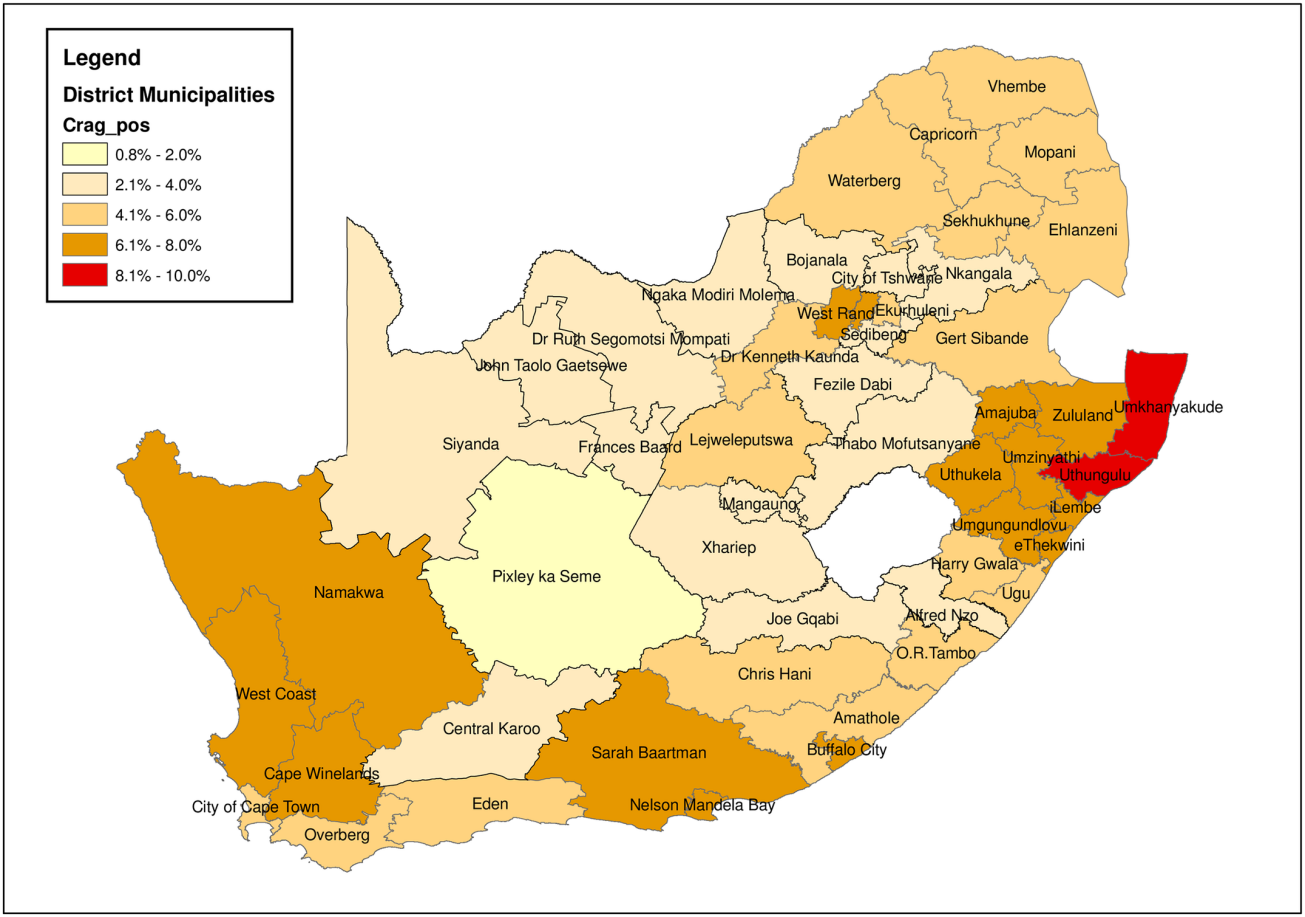
Spatial representation of CrAg positivity. A map of South Africa representing the 52 districts, color coded according to the Crag positivity rates at district level.

The %CrAg positivity is further displayed in Fig 3 (S1 Table) as a map of South Africa defining the location of the 52 districts. Color coding confirmed that the highest CrAg positivity rates were located in KZN districts, i.e. Uthungulu and Umkhanyakude (>8%). Fig 3 further revealed that 1/52 districts had a CrAg positivity between 6–8%, 18/52 had a positivity between 4.1–6%, 16/52 had a positivity between 2.1–4% and a further 5/52 had a positivity of 5.4–6%. Only one district had a CrAg positivity <2% (Pixley ka Seme in the NC, geographically centrally located in South Africa).

## Discussion

Reflexed CrAg screening was successfully rolled out within the NHLS over a 4-month period in the latter half of 2016. Implementation was made possible by the procedures and systems already in place across the network of existing CD4 laboratories, as well as the additional support systems specifically geared toward ease of implementation developed during the pilot phase of the programme in selected CD4 testing facilities [17]. Reflexed CrAg screening was scaled up to monthly test volumes of 24 000 tests from October 2016 onward, with more than 96% of all CD4 samples with a confirmed CD4 count<100 cells/μl receiving a reflexed CrAg test. The test volumes, compliance rate and proportion of CD4 <100 cells/μl (~10%) has been maintained monthly as the programme continues.

The data presented builds on recently published data on the distribution of severely immunosuppressed patients (percentage of number of CD4 tests <100 cells/μl), by district by expanding it to include CrAg positivity and the actual number of CrAg-positive samples tested [8]. The CrAg prevalence study conducted prior to the national CrAg reflex implementation reported a national CrAg prevalence of 3.4%, with the highest prevalence reported for the KZN province (6.5%). A limitation of this preliminary study was that a province was represented by one or 2 laboratories at most and that data were only collected for one week.

Data reported in this studyconfirms clearly demonstrates that there is no correlation between the percentage/proportion of CD4 samples with a count <100 cells/μl reported and CrAg positivity (prevalence) (data not shown, correlation results, Spearman r = -0.31). Previously reported data [8] relied on the proportion of CD4 samples with a count <100 cells/μl only as CrAg positivity data only became available for monitoring after national implementation. As an example, KZN reported the lowest percentage of CD4 samples with counts <100 cells/μl, while at the same time having the highest rate of CrAg prevalence at 7.3%. This emphasizes the importance of using available data to prioritize areas/sites for national implementation of reflexed CrAg screening.

As expected, hospitalized patients presented with very low CD4 counts (median of 37 cell/μl), compared to clinic referrals (median CD4 of 51 cells/μl), reflecting the trend of late presentation with advanced disease in South Africa [8]. Overall, the median CD4 count of all patients with a count <100 cells/μl was 46 cells/μl, in line with the preliminary prevalence data, confirming severe immune-suppression in these patients and their vulnerability to co-infections.

The operational data reported here further confirms that CrAg positivity (as with the % patients with CD4<100 cells/μl) is not geographically uniformly distributed across provinces and 52 districts [8]. Two districts had a CrAg positivity exceeding 8% (both in KZN) with 16 of 52 districts reporting <4% CrAg positivity. Visualizing the relationship between advanced HIV disease/proportion (%CD4 <100 cells/μl) and burden of cryptococcal disease (%CrAg positivity) using geographical mapping identified districts in urgent need of prioritized HIV and cryptococcal disease intervention. Districts with higher than national average CrAg positivity and large numbers of CrAg-positive samples, such as eThekwini and JHB Metro accounted for almost a quarter of all CrAg-positive samples reported for the period of this study. Health services in these districts need to prioritize both ART initiation and optimize management of CrAg-positive patients, including initiation of anti-fungal treatment. Patients with exceptionally low CD4 counts (12% with CD4 count<10cells/μl) need urgent follow up for clinical assessment. Algorithms and systems need to be developed to (ultra) fast-track these patients into care and monitor them for immune reconstitution inflammatory syndrome (IRIS) that may develop during the course of treating their concomitant HIV and cryptococcal disease.

## Conclusion

Existing CD4 testing services were rapidly scaled up to include CrAg reflex testing across a network of laboratories in South Africa and shows that operational data can be used to identify areas with high disease burden. Poor correlation was indicated between the percentage of samples with a CD4 count <100 cells/μl and CrAg positivity at provincial and district levels and neither were uniformly distributed. Prevalence and positivity were fairly stable over time, with minor fluctuations in test volumes following CD4 testing trends. Visualizing CrAg positivity by district highlighted areas for timeous clinical intervention and follow up.

## Limitations

The CD4 laboratory data used in this study were not able to distinguish between first-ever and follow-up CD4 tests, and may include both, due to the absence of a unique patient identifier. The study merely reported operational specimen level data without the ability to generate a cohort analysis for follow up of patients onto anti-fungal treatment.

For the purpose of this study, all reported CrAg and CD4 tests for the period of the study was used (episode number), i.e. operational data. Patient level data (unique patient identification), i.e. de-duplicated data are currently being collected and analyzed for presentation of CrAg prevalence at the one year anniversary of the reflex testing programme. Hence, for this paper the percentage positivity is reported and not prevalence. Preliminary de-duplication of CD4 and CrAg data (not reported) however indicated an impact of <1% on overall reported positivity rate of CrAg in South Africa.

## Supporting information

S1 Table Data summary used for Figs 1 and 2. (XLSX)
